# Supplementary material for: Oral Palatability Testing of a Medium-Chain Triglyceride Oil Supplement (MCT) in a Cohort of Healthy Dogs in a Non-Clinical Setting
Source: Animals (Basel). 2022 Jun 26;12(13):1639. doi: 10.3390/ani12131639 (PMC9264852; doi:10.3390/ani12131639)
Supplement: Supplementary file 1 [file animals-12-01639-s001.zip › animals-1753338-supplementary.pdf]

Phase 1	Referenz
Phase 2	Öl
Phase 3	Nachw

Studie	₹VC-Epi_MCT-1(
Studiennummer	1 01
Termin:	30/11/16
Phase 1 bis:	05/12/16
Phase 2 bis;	10/12/16
Phase 3 bis:	15/12/16

Tag	Datum	FM 1 in g	FM 2 in g	FZ in sec	F	HA	KA	Bemerkungen	Sig
Köpergewicht an Tag 1 (Kilogramm):		___________ kg		Leergewicht der Schüssel (Gramm):				_____________ g	
1	30/11/16								
2	01/12/16								
3	02/12/16								
4	03/12/16								
5	04/12/16								
Köpergewicht an Tag 6 (Kilogramm):				___________ kg					
Ab Tag 6 eine Spritze Öl zur morgendlichen Mahlzeit hinzufügen!									
6	05/12/16								
7	06/12/16								
8	07/12/16								
9	08/12/16								
10	09/12/16								
Köpergewicht an Tag 11 (Kilogramm):				___________ kg					
11	10/12/16								
12	11/12/16								
13	12/12/16								
14	13/12/16								
15	14/12/16								

Figure S1. Feeding diary provided to each owner for record keeping throughout the duration of the study.
